# One-year mortality after recovery from critical illness: A retrospective cohort study

**DOI:** 10.1371/journal.pone.0197226

**Published:** 2018-05-11

**Authors:** Sharukh Lokhandwala, Ned McCague, Abdullah Chahin, Braiam Escobar, Mengling Feng, Mohammad M. Ghassemi, David J. Stone, Leo Anthony Celi

**Affiliations:** 1 Massachussetts Institute of Technology, Cambridge, Massachusetts, United States of America; 2 University of Washington, Division of Pulmonary, Critical Care, and Sleep Medicine, WA, United States of America; 3 Kyruus, Boston, MA, United States of America; 4 Memorial Hospital of Rhode Island, Pawtucket, Rhode Island, United States of America; 5 Escuela de Ingeniería de Antioquia, Envigado, Colombia; 6 Saw Swee Hock School of Public Health, National University of Singapore, Singapore; 7 University of Virginia School of Medicine, Charlottesville, Virginia United States of America; 8 Harvard Medical School, Boston, Massachusetts, United States of America; 9 Beth Israel Deaconess Medical Center, Boston, Massachusetts, United States of America; Azienda Ospedaliero Universitaria Careggi, ITALY

## Abstract

**Rationale:**

Factors associated with one-year mortality after recovery from critical illness are not well understood. Clinicians generally lack information regarding post-hospital discharge outcomes of patients from the intensive care unit, which may be important when counseling patients and families.

**Objective:**

We sought to determine which factors among patients who survived for at least 30 days post-ICU admission are associated with one-year mortality.

**Methods:**

Single-center, longitudinal retrospective cohort study of all ICU patients admitted to a tertiary-care academic medical center from 2001–2012 who survived ≥30 days from ICU admission. Cox’s proportional hazards model was used to identify the variables that are associated with one-year mortality. The primary outcome was one-year mortality.

**Results:**

32,420 patients met the inclusion criteria and were included in the study. Among patients who survived to ≥30 days, 28,583 (88.2%) survived for greater than one year, whereas 3,837 (11.8%) did not. Variables associated with decreased one-year survival include: increased age, malignancy, number of hospital admissions within the prior year, duration of mechanical ventilation and vasoactive agent use, sepsis, history of congestive heart failure, end-stage renal disease, cirrhosis, chronic obstructive pulmonary disease, and the need for renal replacement therapy. Numerous effect modifications between these factors were found.

**Conclusion:**

Among survivors of critical illness, a significant number survive less than one year. More research is needed to help clinicians accurately identify those patients who, despite surviving their acute illness, are likely to suffer one-year mortality, and thereby to improve the quality of the decisions and care that impact this outcome.

## Introduction

As early recognition, effective resuscitation, and technological advancements in the ongoing support of critically ill patients have improved mortality outcomes over recent decades[1–3], there has been growing interest in mortality prediction tools [4–8] in the intensive care unit (ICU) as well as an increasing focus on post-discharge outcomes[9]. One potentially important use for such tools is to reduce, and even avoid, unnecessary and undesirable interventions at the end of life. Among patients who do not survive their ICU admissions, greater than 50% have potentially life-sustaining therapies withheld or withdrawn during that final stay [10, 11]. However, intensivists frequently lack long-term follow up on patients who were “successfully” discharged from the intensive care unit.

Despite clinicians' efforts to determine which patients will have poor outcomes in order to limit futile care, a significant number of patients experience long, complicated clinical courses that are not captured by the current ability of providers to predict one-year mortality. Studies suggest that ICU survivors who die within one year of discharge have a poor quality of life prior to death[12, 13]. Prior studies have demonstrated that those with prolonged mechanical ventilation spend an average of 74% of all days alive post-discharge in a hospital, post-acute care facility or at home but requiring skilled care[12]. Additionally, numerous factors are associated with long term mortality, including ICU length of stay and severe sepsis[13–15]. Rather than dying at home, many deaths occurred either in an acute-care hospital, skilled nursing facility, or long-term care facility with each subsequent hospitalizations within the last year of life being associated with progressive disability and poor quality of life[16].

We therefore sought to determine which factors are associated with one-year mortality among patients who survived critical illness for at least 30 days. Note that our intention is not to categorize a group of patients who may be statically defined as ‘chronically critically ill’ based on ICU length of stay and certain clinical characteristics, but rather to describe the dynamic course of critically ill patients who survive their initial critical illness and then suffer one-year mortality, which prior data suggest is associated with poor quality of life and functional status during the remaining time alive[17]. While there will be some overlap between these two populations, we believe it is essential to point out and clarify this fundamental difference. The ‘chronically critically ill’ as defined by Kahn et al., represent a cohort of patients with particular conditions who have endured a defined period of critical care-the definition does not include further, longer term outcomes. In contrast, the very definition of our short-term survivors rests on two outcomes that combine (live) hospital discharge with one year mortality. We then proceed to attempt to identify the specific conditions and treatments associated with patients eventually included in this group.

## Materials and methods

We conducted a longitudinal, single center, retrospective study of patients who survive at least 30 days from the time of ICU admission to determine which factors are associated with one-year mortality.

Data for this study was collected from the Medical Information Mart for Intensive Care (MIMIC-III) database[18]. All patient data were anonymized prior to extraction and data analysis. The creation, maintenance, and use of the MIMIC-III database was approved by the institutional review boards of the Massachusetts Institute of Technology (MIT: 0403000206) and Beth Israel Deaconess Medical Center (BIDMC: 2001-P-001699). The database contains records from 38,597 adult ICU patients admitted to Boston’s Beth Israel Deaconess Medical Center between 2001–2012. The database includes hourly physiologic readings from bedside monitors, validated by ICU nurses. The database also contains records of demographic information, laboratory results including interpretation of imaging, clinician notes, medications, fluid balance, as well as International Classification of Diseases 9th Revision (ICD-9) and Diagnosis-Related Group (DRG) codes for each hospitalization. Sepsis was defined according to the same ICD-9 criteria as those presented in Angus et al.[19] because MIMIC-III antedates the introduction of sepsis-specific codes into the ICD-9 nomenclature. Most relevant to this project, post-discharge survival is captured using data from the Social Security Death Records, which are updated annually.

### Definition of the cohort

Eligibility was limited to those patients who were over 15 years of age at ICU discharge and survived at least 30 days from the date of ICU admission.

### Outcome measurement

The primary outcome was one-year mortality, measured from the patient’s admission date. For patients with multiple ICU admissions, the most recent admission was included in the analysis. A post-hoc sensitivity analysis was performed using five-year mortality from admission date as the primary outcome.

### Statistical analysis

The baseline characteristics of the population were examined using bivariate analyses for continuous and categorical variables using t-tests and chi-square tests, respectively. Non-parametric methods including the Mann-Whitney U test were used for continuous variables that were not normally distributed, as assessed by Q-Q plots, Kolmogorov–Smirnov tests, and visual inspection of the distributions.

A multivariate Cox’s proportional hazards model was used to assess survival. For our study, survival was censored at one year, and the assumption of proportional hazards was assessed for each variable. No variable failed to meet the assumption of proportional hazards, which was assessed using log-log plots, Kaplan Meier curves, time-dependent covariate assessments, and Schoenfeld residuals. See the online data supplement S1 Text and S1–S23 Figs for descriptions and relevant plots for the included variables.

Candidate variables, which were selected based on previous literature, clinician input, and biologic plausibility, included patient demographics, co-morbidities as captured by ICD-9 diagnoses, interventions received (e.g. mechanical ventilation, use of vasoactive agents, renal replacement therapy, tracheostomy), severity and trajectory of illness as captured by Sequential Organ Failure Assessment (SOFA) scores[20], presence or absence of sepsis, development of acute kidney injury as defined by the Acute Kidney Injury Network [21], hospitalization/ICU admission within the previous year and ICU type. The change in SOFA score was calculated by subtracting the SOFA score from days 3 and 2 from the SOFA score on day 1. Days on ventilation and age were modeled as continuous variables, and the assumption of proportional hazards was validated using Schoenfeld’s residuals in a smoothing cubic spines model. Vasoactive agent use–including norepinephrine, epinephrine, phenylephrine, isoproterenol, dopamine, dobutamine, vasopressin and milrinone–was initially considered as a binary variable, but this did not meet the assumption of proportional hazards. We instead included vasoactive exposure as a continuous variable (i.e. days on vasoactive agents. Urgency of ICU admission (elective, urgent/emergent) was included as a categorical variable. Wald tests were used to assess the impact of two-way interaction terms on a reasonable set of pre-specified, clinically relevant interaction terms. The set of variables that created the interaction terms included ventilation, sepsis, RRT, liver disease, and ESRD.

All reported p-values were rounded to four decimal places. Data were analyzed using SAS version 9.3. All statistical tests and/or confidence intervals, as appropriate, were performed at α = 0.05 (2-sided).

## Results

**Fig 1:**
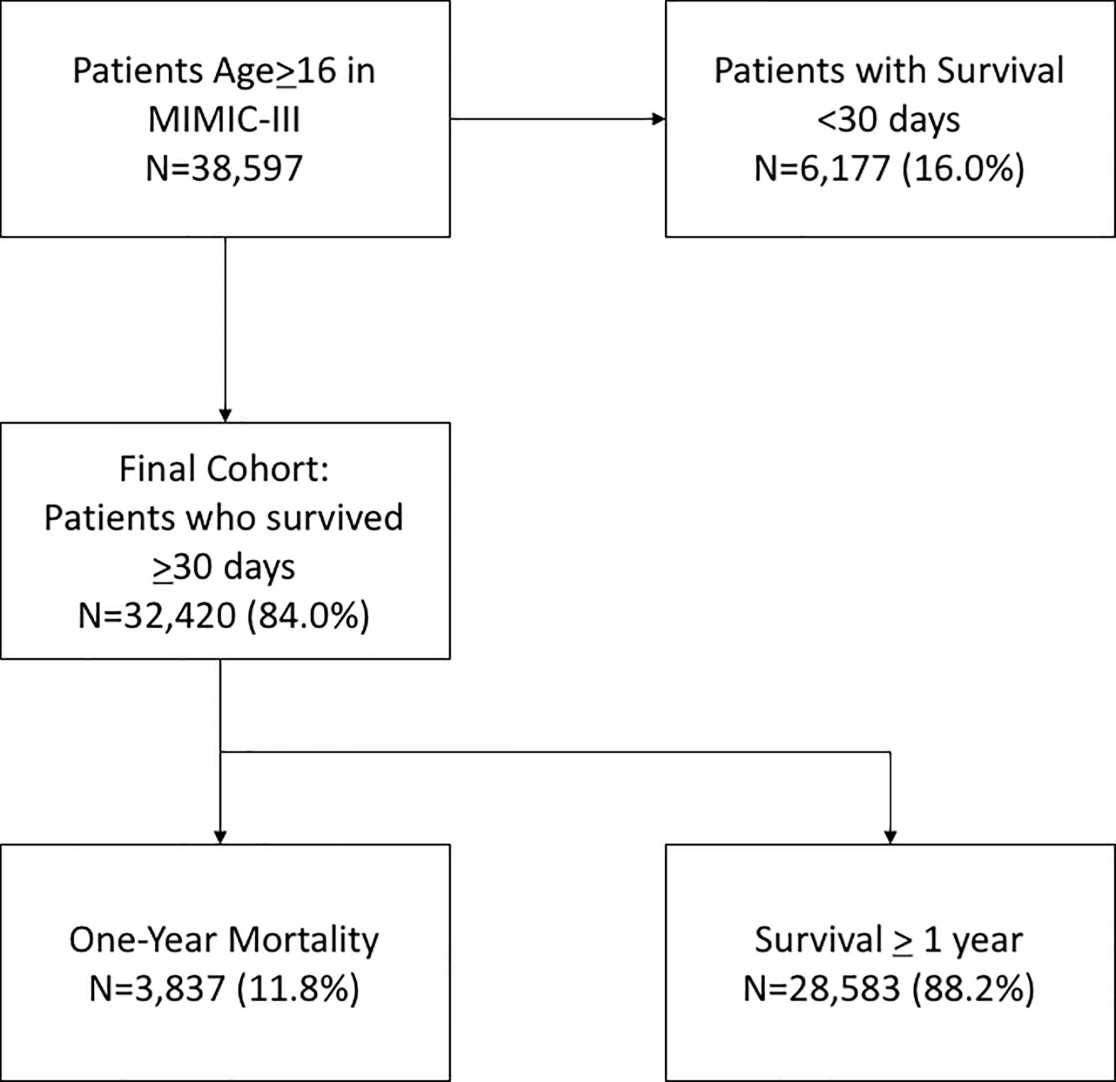
Flow diagram showing initial selection of cohort and excluded patients.

As noted in Fig 1, 32,420 patients who survived at least 30 days after ICU admission were included in the cohort. Of these, 3,837 patients (11.8%) survived less than one year. In univariate analyses, patients with one-year mortality had higher co-morbid rates of congestive heart failure (38.96% vs. 20.16%, p<0.0001), chronic obstructive pulmonary disease (COPD) (20.80% vs. 10.04%, p<0.0001), and end-stage renal disease (ESRD) (4.90% vs. 1.89%, p<0.0001). Those with one-year mortality were more likely to require initiation of hemodialysis (7.51% vs. 3.06%, p<0.0001) and more likely to be diagnosed with sepsis (39.95% vs. 22.36%, p<0.0001). Mechanical ventilation was more common among those who survived greater than one year (43.75% vs. 35.73%, p<0.0001), however the duration of mechanical ventilation was shorter (median duration 1 day vs. 2 days, p<0.0001). See Table 1 for details.

**Table 1:** Comparison based on length of survival *†.

	One-year mortality	Survival > One-year	P-value
**N**	3837 (11.8)	28583 (88.2)	
**Age, years**	72.14	61.22	<0.0001
**Male gender**	2074 (54.05)	16540 (57.87)	<0.0001
**Co-Morbid Conditions**			
Congestive Heart Failure	1495 (38.96)	5763 (20.16)	<0.0001
Cirrhosis	285 (7.43)	1609 (5.63)	<0.0001
Cerebrovascular Accident	179 (4.67)	1424 (4.98)	0.428
COPD	798 (20.80)	2871 (10.04)	<0.0001
ESRD	188 (4.90)	540 (1.89)	<0.0001
Obesity	121 (3.15)	1718 (6.01)	<0.0001
Hypertension	1388 (36.17)	12961(45.35)	<0.0001
Diabetes	1084 (28.25)	6992 (24.46)	<0.0001
**Length of Stay**			
ICU LOS, days	2.28 [1.32–4.34]	1.97 [1.15–3.48]	<0.0001
**Admission within 1 year prior**‡			
Hospital	0.30±0.72	0.12±0.43	<0.0001
ICU	0.39±0.85	0.18±0.54	<0.0001
**SOFA Score**			
Day 1 score	2.47±2.50	2.74±2.71	0.0003
Day 2 score—Day 1 score^**δ**^	-0.53±1.94	-0.98±2.27	<0.0001
Day 3 score—Day 1 score^Ψ^	-0.84±2.39	-1.23±2.76	<0.0001
**ICU Type**			
MICU	1,848 (48.16)	8,901 (31.14)	<0.0001
CCU	626 (16.31)	3,932 (13.76)	<0.0001
CSRU	366 (9.54)	7,226 (25.28)	<0.0001
SICU	677 (17.64)	4,714 (16.49)	0.075
**Comfort Care Order**	694 (18.09)	5,536 (19.37)	0.059
**Mechanical Ventilation**			
Mechanical Ventilation Use	1,371 (35.73)	12,505 (43.75)	<0.0001
Duration of Mechanical Ventilation, days ^Θ^	2 [1–6]	1 [1–3]	<0.0001
**Vasoactive Agent Use**			
Vasoactive Agent Use	674 (17.57)	4,147 (14.51)	<0.0001
Duration of Vasoactive Agent Use, days^Ω^	2 [1–3]	1 [1–3]	<0.0001
**Sepsis**	1,533 (39.95)	6,391 (22.36)	<0.0001
**Cardiac Dysrhythmia**	1,500 (39.09)	7,604 (26.60)	<0.0001
**Coagulopathy**	489 (12.74)	2,335 (8.17)	<0.0001
**Dialysis**	288 (7.51)	876 (3.06)	<0.0001
**Tracheostomy**	272 (7.09)	1,999 (6.99)	0.814

*All continuous variables are expressed as medians [inter quartile range] or mean±SD depending on normality of data. Categorical data is expressed as n (frequency).

†Abbreviations: COPD: Chronic obstructive pulmonary disease; ESRD: End-stage renal disease; LOS: Length of stay; ICU: Intensive care unit; SOFA: Sequential organ failure assessment; MICU: Medical intensive care unit; CCU: Cardiovascular intensive care unit; CSRU: Cardiac surgery intensive care unit; SICU: Surgical intensive care unit

‡Admission (hospital and/or ICU) within 1 year prior to index ICU stay

δ 16,231 observations

Ψ10,304 observations

Θ 13,876 observations

Ω 4,821 observations

Using a Cox proportional hazards model, numerous variables were associated with one-year mortality. The variables most strongly associated with one-year mortality were malignancy (HR = 2.53, p<0.0001), need for renal replacement therapy (RRT) (HR = 1.79, p<0.0001), and urgent/emergent ICU admission (HR = 1.64, p<0.0001). Other factors associated with one-year mortality are ESRD, Cirrhosis, frequency of admissions in the prior year, ICU type, Sepsis, COPD, diabetes, cardiac dysrhythmias, coagulopathy, and the duration of mechanical ventilation. The interaction term RRT*Cirrhosis (HR = 0.51, p = 0.0063) was statistically significantly associated with one-year mortality. Please see online data supplement and Table 2 for further details of the Cox proportional hazards model.

**Table 2:** Cox proportional hazards model for outcome: One-year mortality*.

Variable	Parameter Estimate	Standard Error	Hazard Ratio	95% Confidence Interval	p value
Malignancy	0.929	0.032	2.53	(2.38, 2.69)	< .0001
RRT†	0.582	0.129	1.79	(1.39, 2.31)	< .0001
Admission Type	0.495	0.055	1.64	(1.47, 1.83)	< .0001
ESRD	0.391	0.168	1.48	(1.06, 2.05)	0.0196
Cirrhosis	0.391	0.082	1.48	(1.26, 1.74)	< .0001
COPD	0.375	0.037	1.45	(1.35, 1.57)	< .0001
Hospital Readmission§	0.347	0.043	1.41	(1.30, 1.54)	< .0001
Sepsis	0.304	0.036	1.35	(1.26, 1.45)	< .0001
ICU Type	0.249	0.017	1.28	(1.24, 1.33)	< .0001
Diabetes	0.243	0.059	1.28	(1.13, 1.43)	< .0001
Coagulopathy	0.161	0.046	1.17	(1.07, 1.29)	0.0005
Cardiac Dysrhythmia	0.127	0.033	1.14	(1.07, 1.21)	0.0001
ESRD*Cirrhosis‡	0.064	0.338	1.07	(0.55, 2.07)	0.8487
ICU Readmissions§	0.037	0.027	1.04	(0.98, 1.09)	0.18
Age	0.034	0.001	1.03	(1.03, 1.04)	< .0001
Cerebrovascular Accident	0.026	0.069	1.03	(0.90, 1.17)	0.7035
Duration of Mechanical Ventilation	0.021	0.003	1.02	(1.02, 1.03)	< .0001
Duration of Vasoactive Agent Use	0.007	0.008	1.01	(0.99, 1.02)	0.3911
Male Gender	0.007	0.031	1.01	(0.95, 1.07)	0.8303
Day 1 SOFA Score	-0.021	0.007	0.98	(0.97, 0.99)	0.0012
RRT*Sepsis	-0.155	0.150	0.86	(0.64, 1.15)	0.2989
Sepsis*Cirrhosis‡	-0.176	0.116	0.84	(0.67, 1.05)	0.1294
Comfort Care Order	-0.218	0.041	0.8	(0.74, 0.87)	< .0001
ESRD*Sepsis	-0.226	0.177	0.8	(0.56, 1.13)	0.2029
ESRD*RRT‡	-0.356	0.183	0.7	(0.49, 1.00)	0.0519
Hypertension	-0.409	0.033	0.66	(0.62, 0.71)	< .0001
RRT*Cirrhosis‡	-0.676	0.247	0.51	(0.31, 0.83)	0.0063

*Abbreviations: COPD: Chronic obstructive pulmonary disease; ESRD: End-stage renal disease; LOS: Length of stay; ICU: Intensive care unit; SOFA: Sequential organ failure assessment; MICU: Medical intensive care unit; MV: Mechanical Ventilation

†New initiation of Renal Replacement Therapy in the ICU

§Admission within 1 year of index ICU stay

‡Interaction terms

We additionally performed a post-hoc analysis using five-year mortality as the outcome of a Cox proportional hazards model. RRT (HR = 2.13, p<0.0001), malignancy (HR = 1.95, p<0.0001), ESRD (HR = 1.62, p<0.0001), COPD (HR = 1.53, p<0.0001) and Cirrhosis (HR = 1.52, p<0.0001) were most highly associated with five-year mortality. Please see the S1 Table for details.

## Discussion

Our study identified numerous acute diagnoses and comorbidities associated with one-year mortality after initial survival of critical illness. The presence of malignancy, need for renal replacement therapy and urgent/emergent admission are most strongly associated with one-year mortality among 30-day survivors. Additionally, ESRD, sepsis, COPD, Cirrhosis, Diabetes, prior hospital admissions, and duration of mechanical ventilation are all associated with one-year mortality among 30-day survivors. As expected, many of the variables associated with one-year mortality are likewise associated with five-year mortality in our proportional hazards model.

Additionally, those admitted to a MICU had lower one-year survival as compared to patients admitted to other ICU types (CCU, CSRU, or SICU). We postulate that patients with numerous co-morbidities and higher level of illness are less likely to be placed in specialty-specific intensive care units. In addition, due to their underlying medical problems, these patients may have worse prognoses independent of ICU admission, and/or subsequently be more physiologically compromised by the impact of such admissions.

As noted in previous studies, many patients have care withdrawn or withheld, and physicians use numerous variables in their determinations regarding whether to limit therapy[22]. Given the significant association with certain ICU interventions (renal replacement therapy, mechanical ventilation duration, vasopressor duration) as well as co-morbid illnesses (cirrhosis, ESRD) with one-year mortality, we endorse that discussions should address the possibility of one-year mortality even if the patient were to overcome critical illness, with Cox and Curtis describing methods with which this can be integrated into the electronic health record[23].

In addition to establishing numerous comorbidities and disease states associated with one-year mortality, we also identified effect-modification between the use of renal replacement therapy and cirrhosis. Effect measure modification is the biological phenomenon in which a certain exposure has a different magnitude of effect in the presence of another exposure. Interaction between the effects of numerous patient-, disease- and treatment-related factors is challenging for clinicians to quantify objectively without computational assistance.

As the care of patients becomes more complex, physicians struggle to determine which patients are likely to suffer a poor quality of life and/or death after surviving critical illness, and little follow up is routinely available to ICU clinicians. Current ICU prediction tools rarely apply to patients after the current hospital stay and do not address longer clinical courses such as the one-year interval that we examined[24, 25]. Our findings should alert clinicians to the relevance of events beyond the immediate scope of the ICU stay and to consider post-ICU outcomes in day-to-day decision-making. This awareness should lead to an ability to conduct more informative and comprehensive discussions with patients and their families regarding the goals of care and the ongoing use or additional implementation of potentially futile medical interventions. Our findings are consistent with those found by Garland, et al., providing more evidence that acute ICU interventions and diagnoses along with co-morbid illnesses are associated with long-term survival among survivors of critical illness[9].

There are several limitations to our study. Our study is limited by its retrospective nature as well as by being from a single, tertiary-care academic center, and our results may therefore not be widely generalizable. Furthermore, potential unmeasured confounders not included in our analysis may also be present. Disease associations based on ICD-9 codes are limited by the reporting of the treating physician(s). Our inclusion of admissions within the prior year only capture those at our single institution, and thus may miss hospitalizations at other institutions. Additionally, we did not adjust for any treatment-related or technological advancements that may have occurred between 2001 and 2012. Furthermore, we chose to focus on post-discharge mortality among 30-day survivors rather than all patients admitted to the intensive care unit. We focused our post-discharge mortality predictions specifically on 30-day survivors rather than all ICU patients for 2 reasons. First, this approach excludes in-hospital mortality within that time window. Given that some individuals might have lived if care had not been limited or terminated, including these individuals could have led to classification error. The 30-day survivor cohort is also roughly equivalent to that of hospital survivors while providing a long post hospital discharge time window to observe mortality rates until the defined limit of one year. Second, one-year mortality among patients who are discharged after an ICU admission may reflect, to some degree, poor prognostication on the part of the clinician: these patients were thought to have potentially positive post-discharge outcomes, otherwise care would presumably have been limited or withdrawn.

Lastly, our main outcome was one-year mortality. There are significant outcomes of value that were not measured, including hospital re-admission, time spent in a facility, and post-discharge utilization of resources. We hope to integrate the Massachusetts All Payer Claims Database[26] into future versions of MIMIC to better characterize post-discharge utilization among critically-ill patients.

## Conclusion

Among survivors of critical illness, a significant proportion survive less than one year. Numerous factors are associated with one-year mortality. More research is needed to help clinicians accurately predict which patients who, despite surviving their acute illness, are likely to suffer one-year mortality. Identification of these factors will aid in the development of clinical decision support tools to assist in the complex, real time therapeutic decisions required in this difficult context and provide feedback to clinicians regarding the long-term outcomes of patients who survive their ICU stay.

## Supporting information

S1 Table Cox proportional hazards model for outcome: Five-year mortality. (DOCX)

S1 Text Supplementary information. (DOCX)

S1 Fig Log-log plot of survival as a function of hospital readmissions. (PNG)

S2 Fig Kaplan Meier plot of survival as a function of hospital readmissions. (PNG)

S3 Fig Log-log plot of survival as a function of sepsis. (PNG)

S4 Fig Kaplan Meier plot of survival as a function of sepsis. (PNG)

S5 Fig Log-log plot of survival as a function of arrhythmia. (PNG)

S6 Fig Kaplan Meier plot of survival as a function of arrhythmia. (PNG)

S7 Fig Log-log plot of survival as a function of cirrhosis. (PNG)

S8 Fig Kaplan Meier plot of survival as a function of cirrhosis. (PNG)

S9 Fig Log-log plot of survival as a function of COPD. (PNG)

S10 Fig Kaplan Meier plot of survival as a function of COPD. (PNG)

S11 Fig Log-log plot of survival as a function of diabetes. (PNG)

S12 Fig Kaplan Meier plot of survival as a function of diabetes. (PNG)

S13 Fig Log-log plot of survival as a function of renal replacement therapy. (PNG)

S14 Fig Kaplan Meier plot of survival as a function of renal replacement therapy. (PNG)

S15 Fig Log-log plot of survival as a function of end stage renal disease. (PNG)

S16 Fig Kaplan Meier plot of survival as a function of end stage renal disease. (PNG)

S17 Fig Log-log plot of survival as a function of ICU type. (PNG)

S18 Fig Kaplan Meier plot of survival as a function of ICU type. (PNG)

S19 Fig Log-log plot of survival as a function of malignancy. (PNG)

S20 Fig Kaplan Meier plot of survival as a function of malignancy. (PNG)

S21 Fig Schoenfeld’s residual plot of duration of mechanical ventilation. (PNG)

S22 Fig Schoenfeld’s residual plot of duration of mechanical ventilation using cubic splines and smoothing. (PNG)

S23 Fig Schoenfeld’s residual plot of duration of mechanical ventilation using cubic splines without smoothing. (PNG)

## Abbreviations

BIDMC: Beth Israel Deaconess Medical Center
COPD: Chronic obstructive pulmonary disease
DRG: Diagnosis-related group
ESRD: End-stage renal disease
LOS: Length of stay
ICD-9: International Classification of Diseases 9th Revision
ICU: Intensive care unit
SOFA: Sequential organ failure assessment
MICU: Medical intensive care unit
MIMIC-III: Medical Information Mart for Intensive Care
MV: Mechanical Ventilation
SOFA: Sequential Organ Failure Assessment
