# Supplementary material for: One-year mortality after recovery from critical illness: A retrospective cohort study
Source: PLoS One. 2018 May 11;13(5):e0197226. doi: 10.1371/journal.pone.0197226 (PMC5947984; doi:10.1371/journal.pone.0197226)
Supplement: S1 Table — (DOCX) [file pone.0197226.s001.docx]

**Table S1:** Cox proportional hazards model for outcome: Five-year mortality*

| **Variable** | **Parameter Estimate** | **Standard Error** | **Hazard Ratio** | **95% Confidence Interval** | **p value** |
| --- | --- | --- | --- | --- | --- |
| RRT† | 0.754 | 0.087 | 2.13 | (1.79, 2.52) | <.0001 |
| Malignancy | 0.668 | 0.023 | 1.95 | (1.86, 2.04) | <.0001 |
| ESRD | 0.481 | 0.123 | 1.62 | (1.27, 2.06) | <.0001 |
| COPD | 0.427 | 0.027 | 1.53 | (1.45, 1.62) | <.0001 |
| Cirrhosis | 0.420 | 0.058 | 1.52 | (1.36, 1.70) | <.0001 |
| Diabetes | 0.261 | 0.043 | 1.3 | (1.19, 1.41) | <.0001 |
| Admission Type | 0.252 | 0.035 | 1.29 | (1.20, 1.38) | <.0001 |
| ESRD*Cirrhosis‡ | 0.248 | 0.234 | 1.28 | (0.81, 2.03) | 0.2882 |
| ICU Type | 0.209 | 0.012 | 1.23 | (1.20, 1.26) | <.0001 |
| Sepsis | 0.200 | 0.026 | 1.22 | (1.16, 1.29) | <.0001 |
| ICU Readmissions | 0.147 | 0.019 | 1.16 | (1.11, 1.20) | <.0001 |
| Hospital Readmission | 0.106 | 0.033 | 1.11 | (1.04, 1.19) | 0.0013 |
| Coagulopathy | 0.097 | 0.035 | 1.1 | (1.03, 1.18) | 0.0053 |
| ESRD*Sepsis‡ | 0.095 | 0.129 | 1.1 | (0.85, 1.42) | 0.4619 |
| Cardiac Dysrhythmia | 0.065 | 0.024 | 1.07 | (1.02, 1.12) | 0.0059 |
| Male Gender | 0.055 | 0.022 | 1.06 | (1.01, 1.10) | 0.0121 |
| Age | 0.038 | 0.001 | 1.04 | (1.04, 1.04) | <.0001 |
| Stroke | 0.041 | 0.047 | 1.04 | (0.95, 1.14) | 0.3802 |
| Duration of Mechanical Ventilation | 0.023 | 0.002 | 1.02 | (1.02, 1.03) | <.0001 |
| Duration of Vasoactive Agent Use | 0.002 | 0.007 | 1 | (0.99, 1.02) | 0.7436 |
| Day 1 SOFA Score | -0.018 | 0.005 | 0.98 | (0.97, 0.99) | 0.0002 |
| Sepsis*Cirrhosis‡ | -0.256 | 0.086 | 0.77 | (0.65, 0.92) | 0.0028 |
| Hypertension | -0.357 | 0.023 | 0.7 | (0.67, 0.73) | <.0001 |
| RRT*Sepsis‡ | -0.429 | 0.107 | 0.65 | (0.53, 0.80) | <.0001 |
| Comfort Care Order | -0.463 | 0.032 | 0.63 | (0.59, 0.67) | <.0001 |
| ESRD*RRT‡ | -0.752 | 0.131 | 0.47 | (0.36, 0.61) | <.0001 |
| RRT*Cirrhosis‡ | -0.974 | 0.190 | 0.38 | (0.26, 0.55) | <.0001 |

*Abbreviations: COPD: Chronic obstructive pulmonary disease; ESRD: End-stage renal disease; LOS: Length of stay; ICU: Intensive care unit; SOFA: Sequential organ failure assessment; MICU: Medical intensive care unit; MV: Mechanical Ventilation

†New initiation of Renal Replacement Therapy in the ICU

§Admission within 1 year of index ICU stay

‡Interaction terms
