# Supplementary material for: One-year mortality after recovery from critical illness: A retrospective cohort study
Source: PLoS One. 2018 May 11;13(5):e0197226. doi: 10.1371/journal.pone.0197226 (PMC5947984; doi:10.1371/journal.pone.0197226)
Supplement: S1 Text — (DOCX) [file pone.0197226.s002.docx]

Data Supplement

One-year mortality after recovery from critical illness: a retrospective cohort study

*Lokhandwala, *McCague, Chahin, Escobar, Feng, Ghassemi, Stone, Celi

*Co-first authors

1. **The relationship of Hospital Readmissions with Survival**

The Kaplan-Meier and log-log plots for Hospital Readmissions in the preceding year (Figures S1 and S2) are shown below. Hospital readmissions was stratified into three groups: those with no readmissions, those with one readmission, and those with greater than one admission in the preceding year.

1. **The relationship of Sepsis with Survival**

The Kaplan-Meier and log-log plots for Sepsis (Figures S3 and S4) are shown below. Sepsis was defined using the Angus Criteria as cited in the manuscript.

1. **The relationship of Arrhythmia with Survival**

The Kaplan-Meier and log-log plots for arrhythmia (Figures S5 and S6) are shown below.

1. **The relationship of Cirrhosis with Survival**

The Kaplan-Meier and log-log plots for cirrhosis (Figures S7 and S8) are shown below.

1. **The relationship of COPD with Survival**

The Kaplan-Meier and log-log plots for COPD (Figures S9 and S10) are shown below.

1. **The relationship of Diabetes with Survival**

The Kaplan-Meier and log-log plots for Diabetes (Figures S11 and S12) are shown below.

1. **The relationship of Renal Replacement Therapy with Survival**

The Kaplan-Meier and log-log plots for Renal Replacement Therapy (Figures S13 and S14) are shown below.

1. **The relationship of End-Stage Renal Disease with Survival**

The Kaplan-Meier and log-log plots for End-Stage Renal Disease (Figures S15 and S16) are shown below.

1. **The relationship of ICU Type with Survival**

The Kaplan-Meier and log-log plots for ICU Type (Figures S17 and S18) are shown below. ICU Type was stratified into four groups: Cardiac Surgery ICU (CSRU), Cardiac Care Unit (CCU), Surgical ICU (SICU) and Medical ICU (MICU).

1. **The relationship of Malignancy with Survival**

The Kaplan-Meier and log-log plots for Malignancy (Figures S19 and S20) are shown below.

1. **The relationship of Duration of Mechanical Ventilation with Survival**

The duration of mechanical ventilation was initially coded as an ordinal variable with levels corresponding to durations of 0, >0-1, >1-2, >2-4, or >4 days. However, the use of this coding criteria violated proportional hazards. We then attempted to stratify duration of mechanical ventilation using durations of 0, >0-3, >3-7, >7-14, or >14 days. However, the use of this coding criteria also violated proportional hazards. We hypothesized that categorical stratification of ventilator days using clinically informed cutoffs violated proportional hazards due to the harvesting effect, and thus used it as a continuous variable.

We sought to validate the duration of mechanical ventilation as a continuous variable using Schoenfeld’s residuals. A plot of the Schoenfeld’s residuals (Figure S21) as well as a fitted curve using smoothing cubic splines (Figure S22) and a fitted curve using cubic splines without smoothing (Figure S23) demonstrate the assumption of proportional hazards. Using this model, duration of mechanical ventilation had a hazard ratio of 1.026 (DF=1, Chi-square 86.31, p<0.001) and was included in the final Cox proportional hazards model.

**Figure S1:** Log-log plot of Survival as a function of Hospital Readmissions

**Figure S2:** Kaplan Meier plot of Survival as a function of Hospital Readmissions

**Figure S3:** Log-log plot of Survival as a function of Sepsis

**Figure S4:** Kaplan Meier plot of Survival as a function of Sepsis

**Figure S5:** Log-log plot of Survival as a function of Arrhythmia

**Figure S6:** Kaplan Meier plot of Survival as a function of Arrhythmia

**Figure S7:** Log-log plot of Survival as a function of Cirrhosis

**Figure S8:** Kaplan Meier plot of Survival as a function of Cirrhosis

**Figure S9:** Log-log plot of Survival as a function of COPD

**Figure S10:** Kaplan Meier plot of Survival as a function of COPD

**Figure S11:** Log-log plot of Survival as a function of Diabetes

**Figure S12:** Kaplan Meier plot of Survival as a function of Diabetes

**Figure S13:** Log-log plot of Survival as a function of Renal Replacement Therapy

**Figure S14:** Kaplan Meier plot of Survival as a function of Renal Replacement Therapy

**Figure S15:** Log-log plot of Survival as a function of End Stage Renal Disease

**Figure S16:** Kaplan Meier plot of Survival as a function of End Stage Renal Disease

**Figure S17:** Log-log plot of Survival as a function of ICU Type

**Figure S18:** Kaplan Meier plot of Survival as a function of ICU Type

**Figure S19:** Log-log plot of Survival as a function of Malignancy

**Figure S20:** Kaplan Meier plot of Survival as a function of Malignancy

**Figure S21:** Schoenfeld’s residual plot of duration of mechanical ventilation

**Figure S22:** Schoenfeld’s residual plot of duration of mechanical ventilation using cubic splines and smoothing

**Figure S23:** Schoenfeld’s residual plot of duration of mechanical ventilation using cubic splines without smoothing
