# Supplementary figures and images for: One-year mortality after recovery from critical illness: A retrospective cohort study
Source: PLoS One. 2018 May 11;13(5):e0197226. doi: 10.1371/journal.pone.0197226 (PMC5947984; doi:10.1371/journal.pone.0197226)

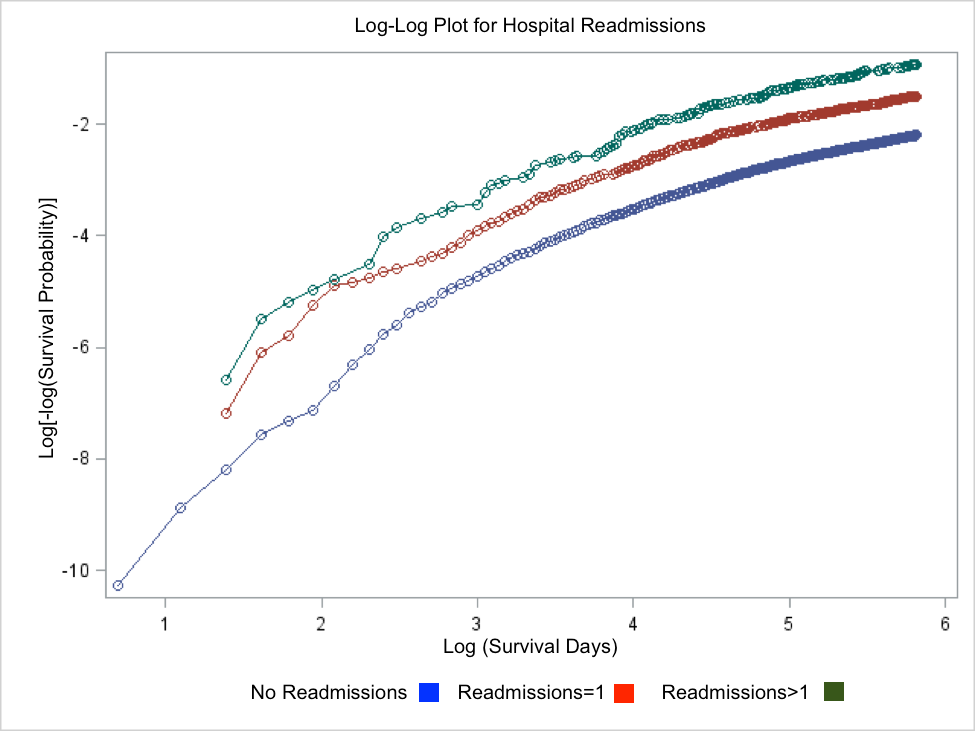

Supplement: S1 Fig — (PNG) [file pone.0197226.s003.png]

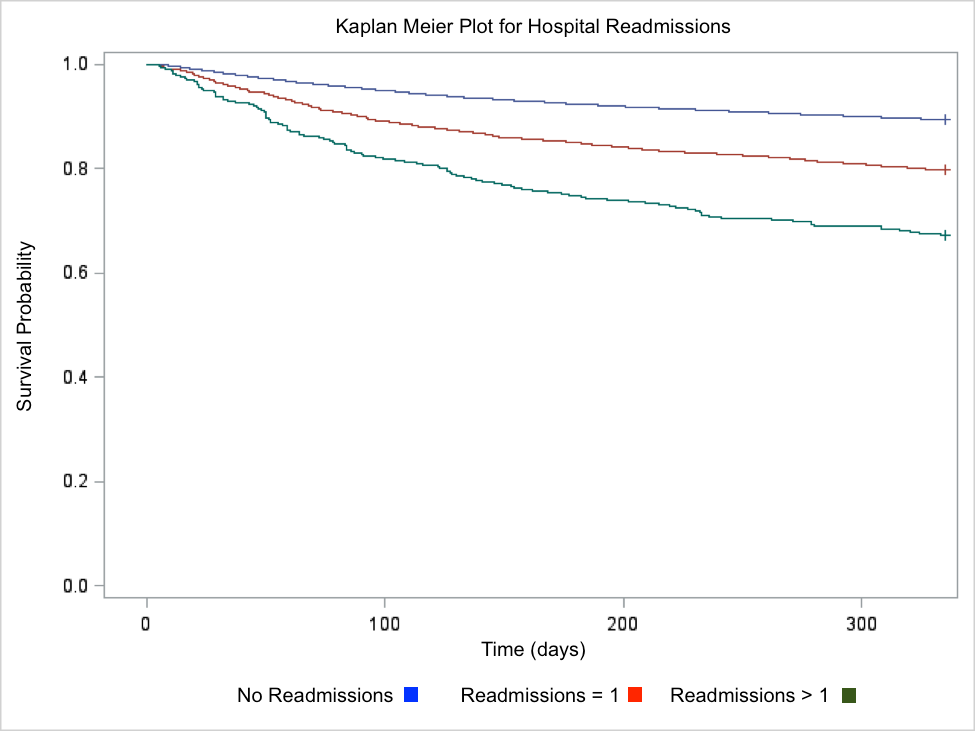

Supplement: S2 Fig — (PNG) [file pone.0197226.s004.png]

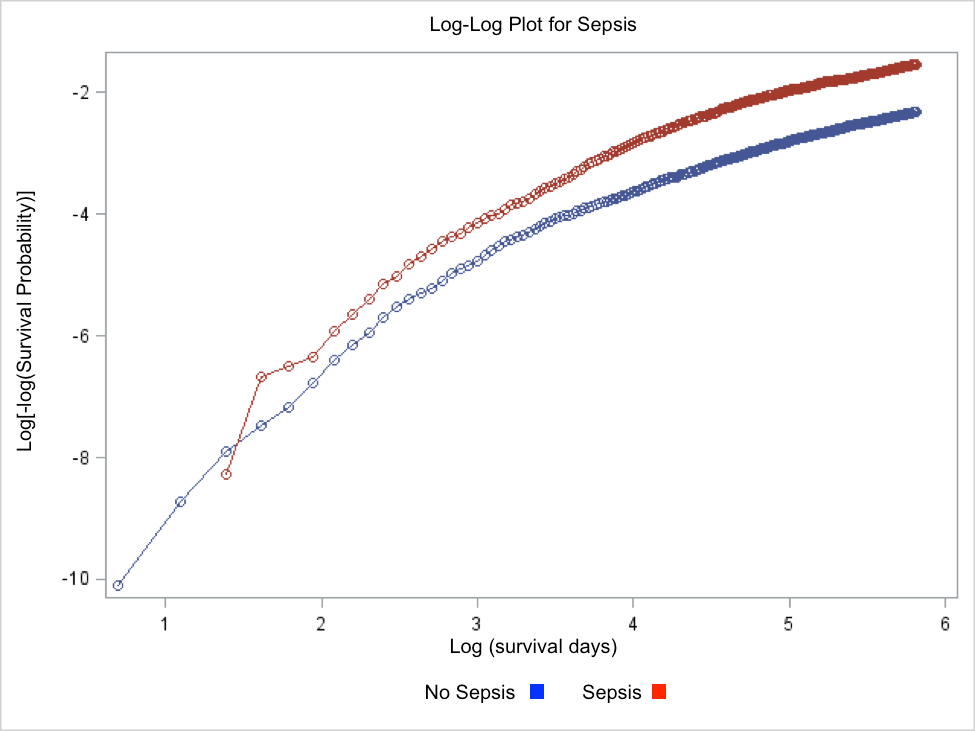

Supplement: S3 Fig — (PNG) [file pone.0197226.s005.png]

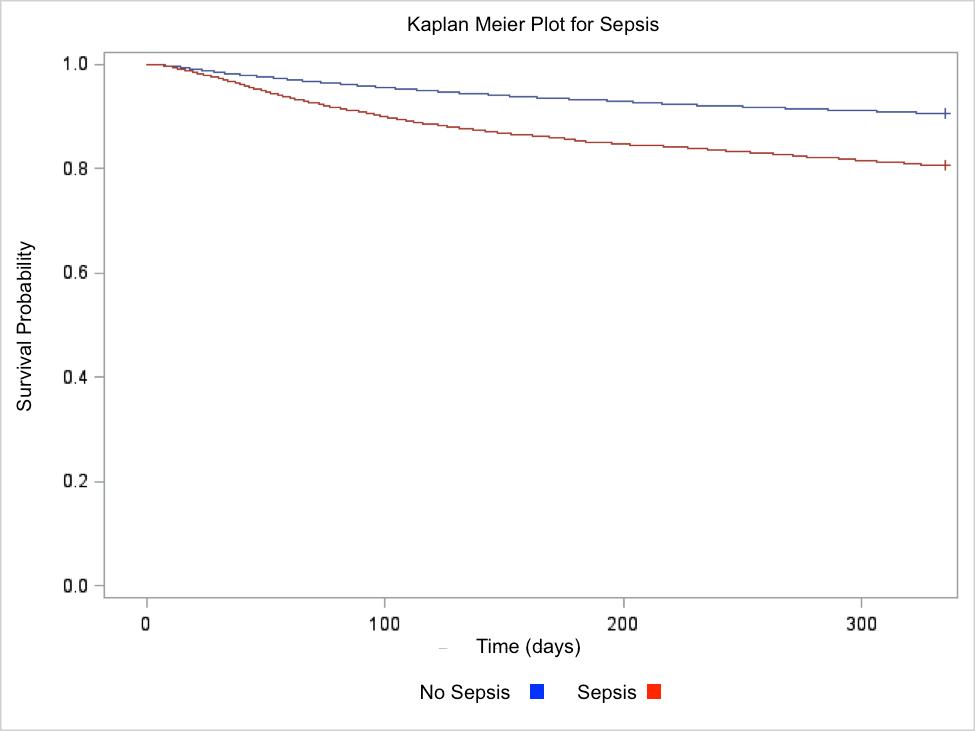

Supplement: S4 Fig — (PNG) [file pone.0197226.s006.png]

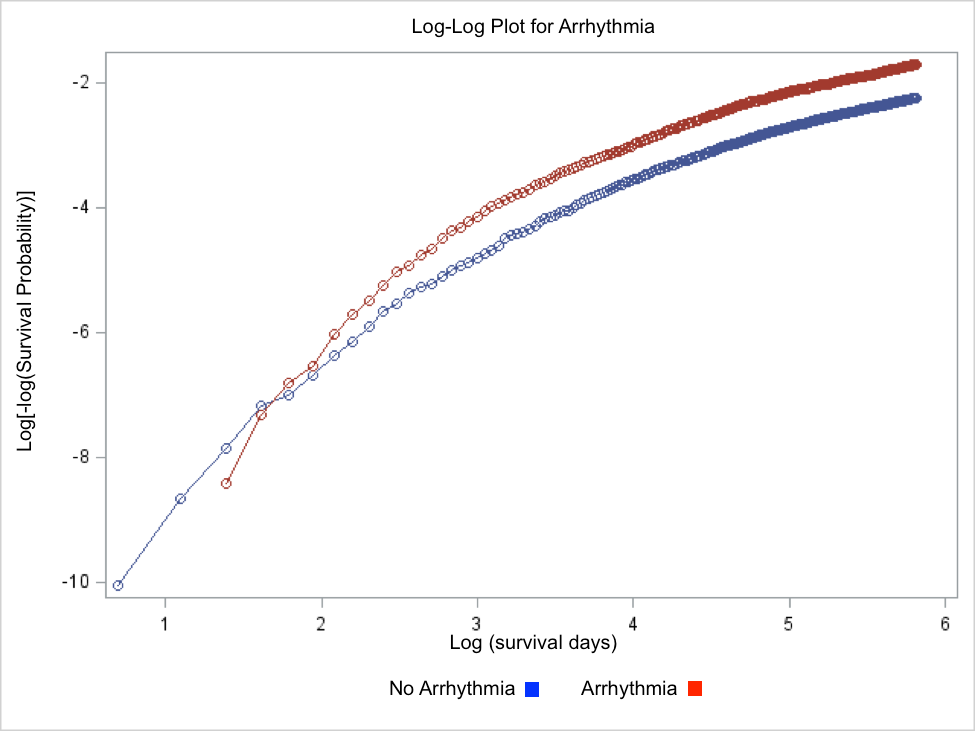

Supplement: S5 Fig — (PNG) [file pone.0197226.s007.png]

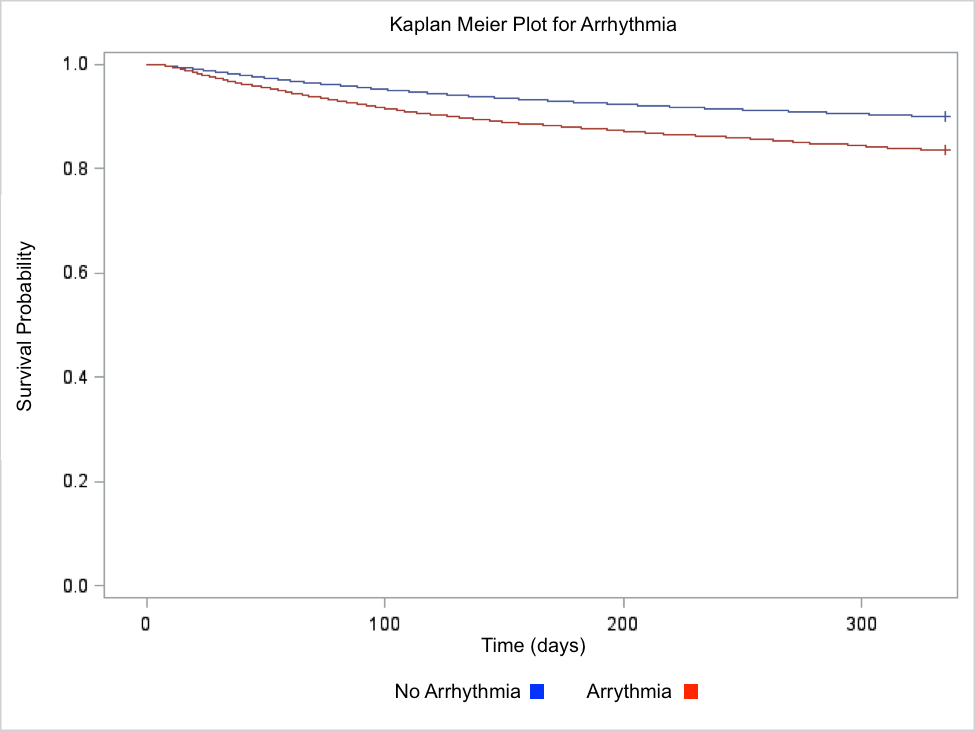

Supplement: S6 Fig — (PNG) [file pone.0197226.s008.png]

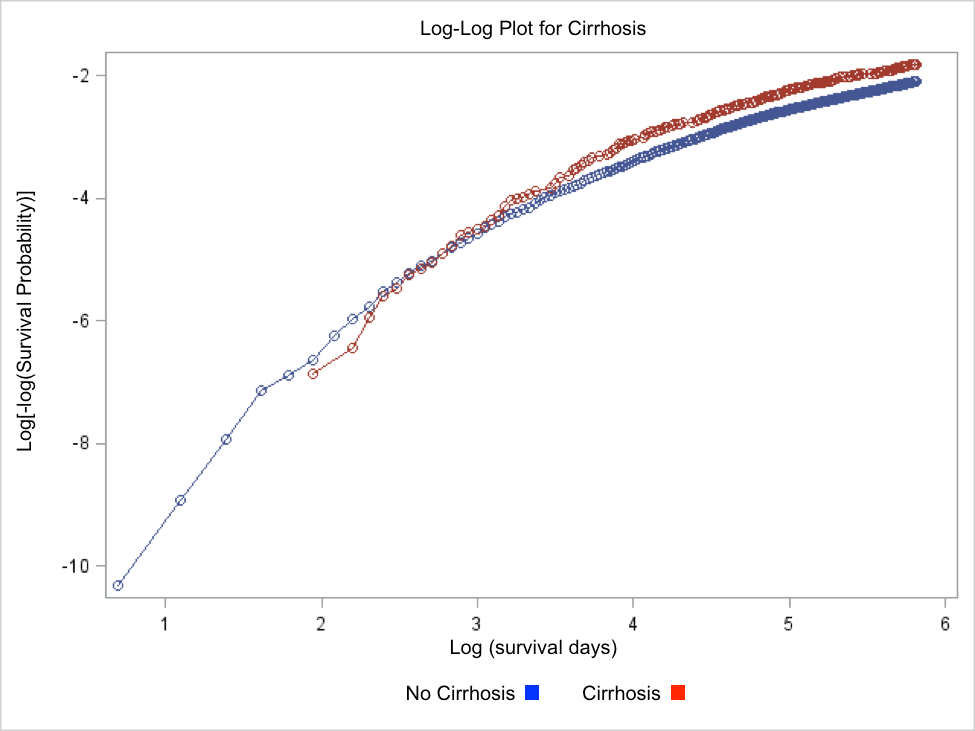

Supplement: S7 Fig — (PNG) [file pone.0197226.s009.png]

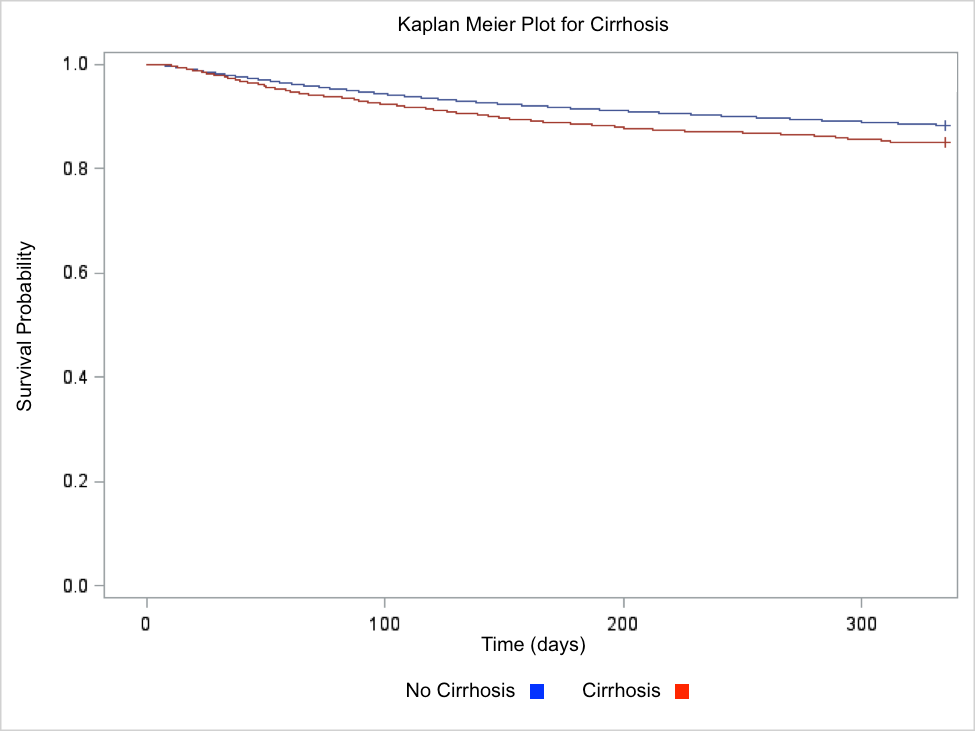

Supplement: S8 Fig — (PNG) [file pone.0197226.s010.png]

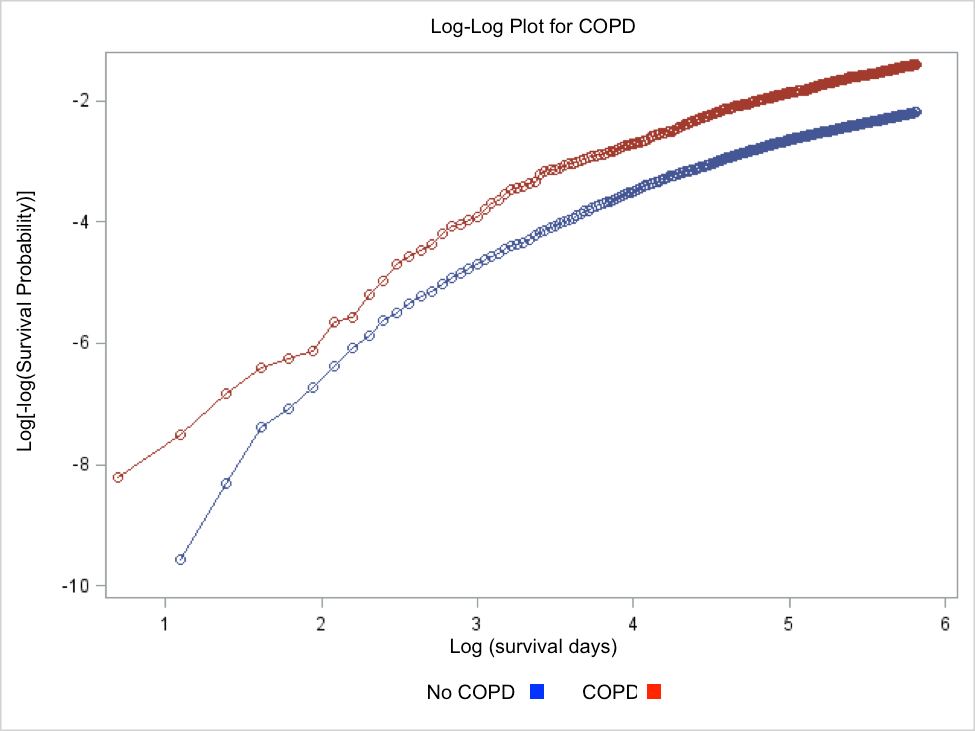

Supplement: S9 Fig — (PNG) [file pone.0197226.s011.png]

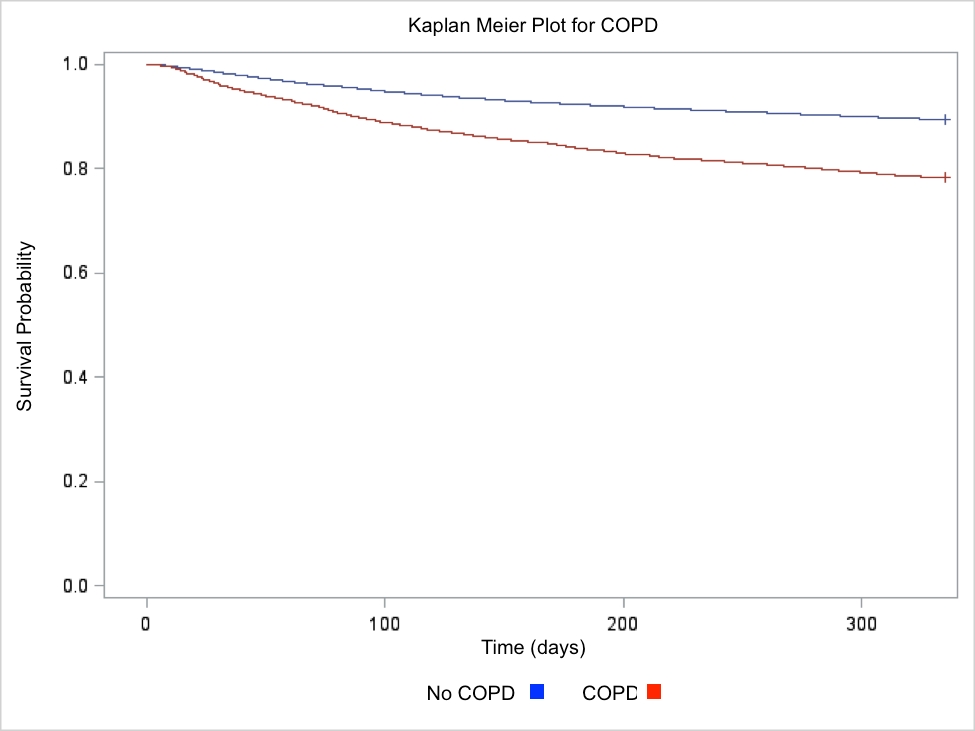

Supplement: S10 Fig — (PNG) [file pone.0197226.s012.png]

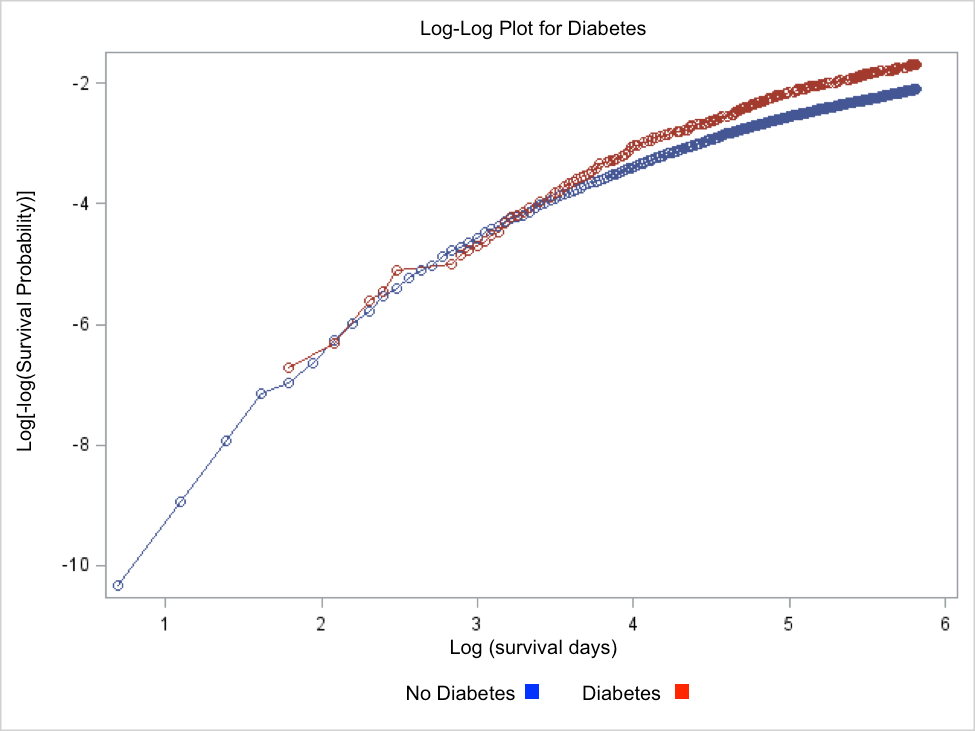

Supplement: S11 Fig — (PNG) [file pone.0197226.s013.png]

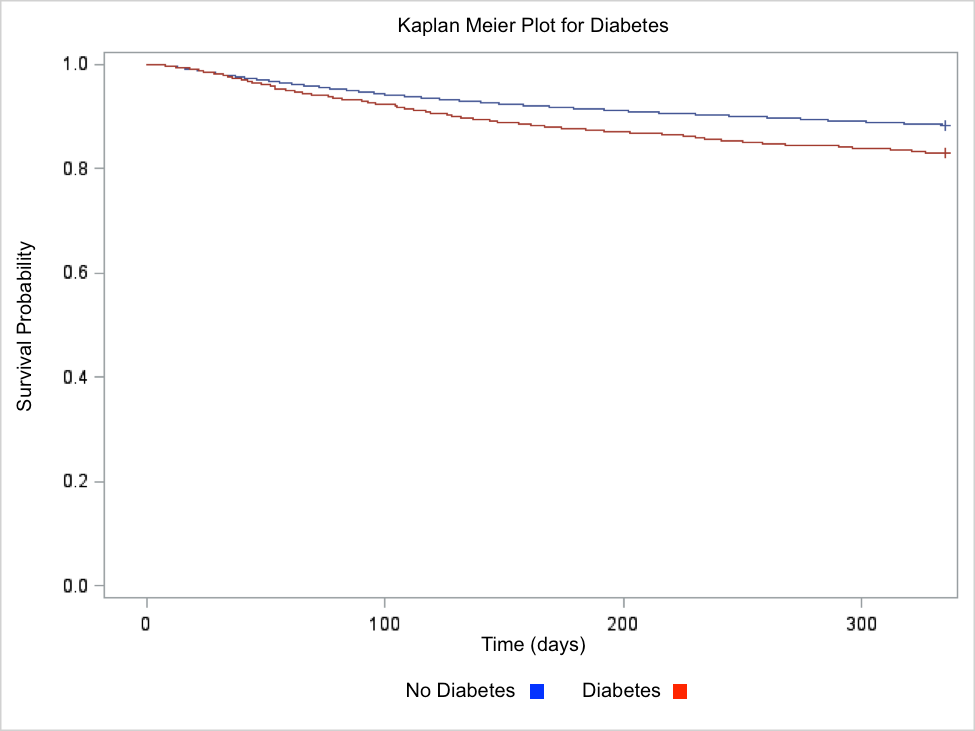

Supplement: S12 Fig — (PNG) [file pone.0197226.s014.png]

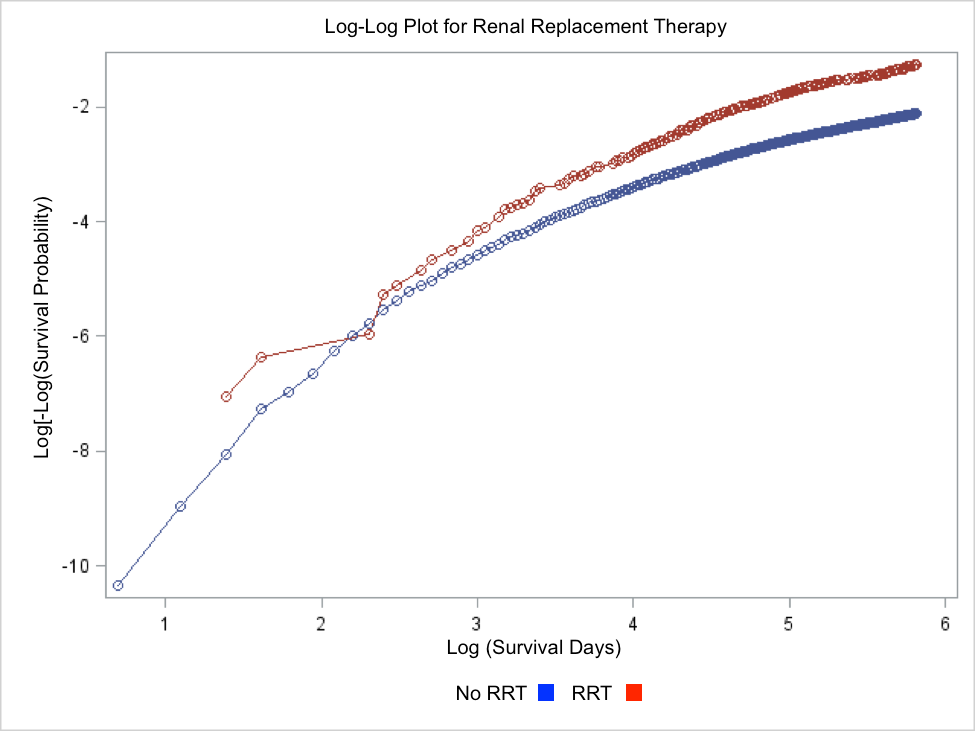

Supplement: S13 Fig — (PNG) [file pone.0197226.s015.png]

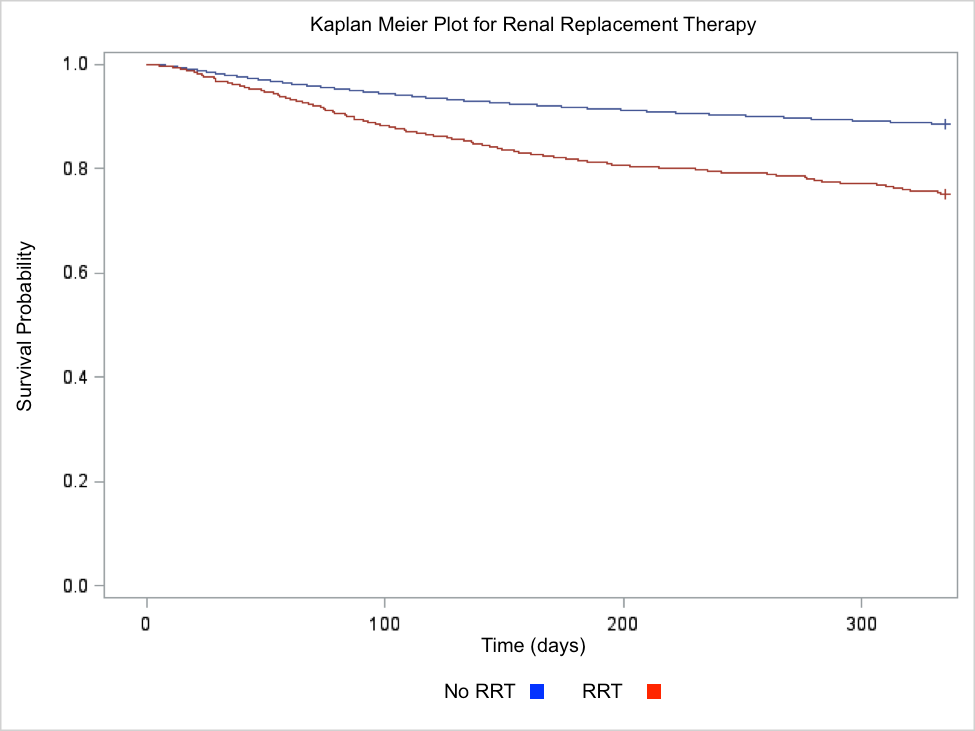

Supplement: S14 Fig — (PNG) [file pone.0197226.s016.png]

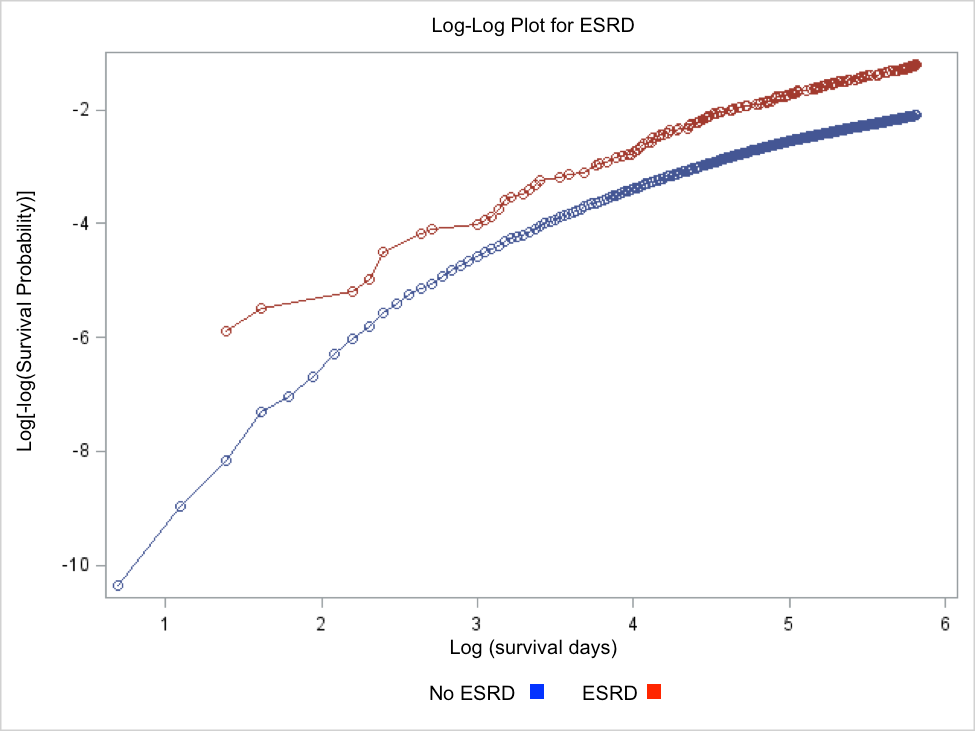

Supplement: S15 Fig — (PNG) [file pone.0197226.s017.png]

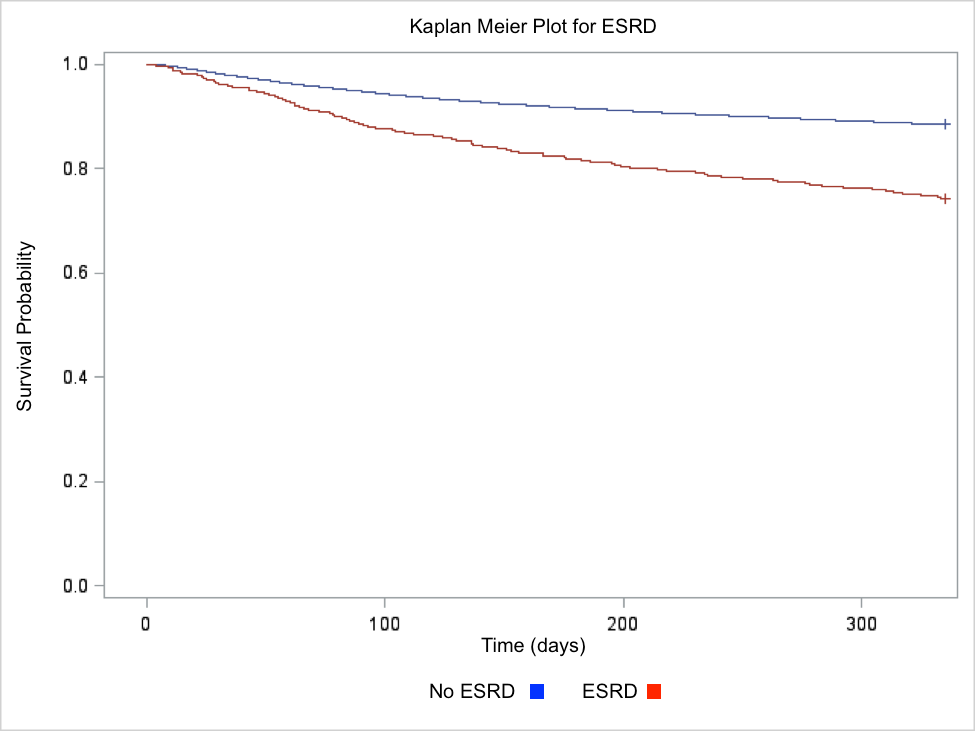

Supplement: S16 Fig — (PNG) [file pone.0197226.s018.png]

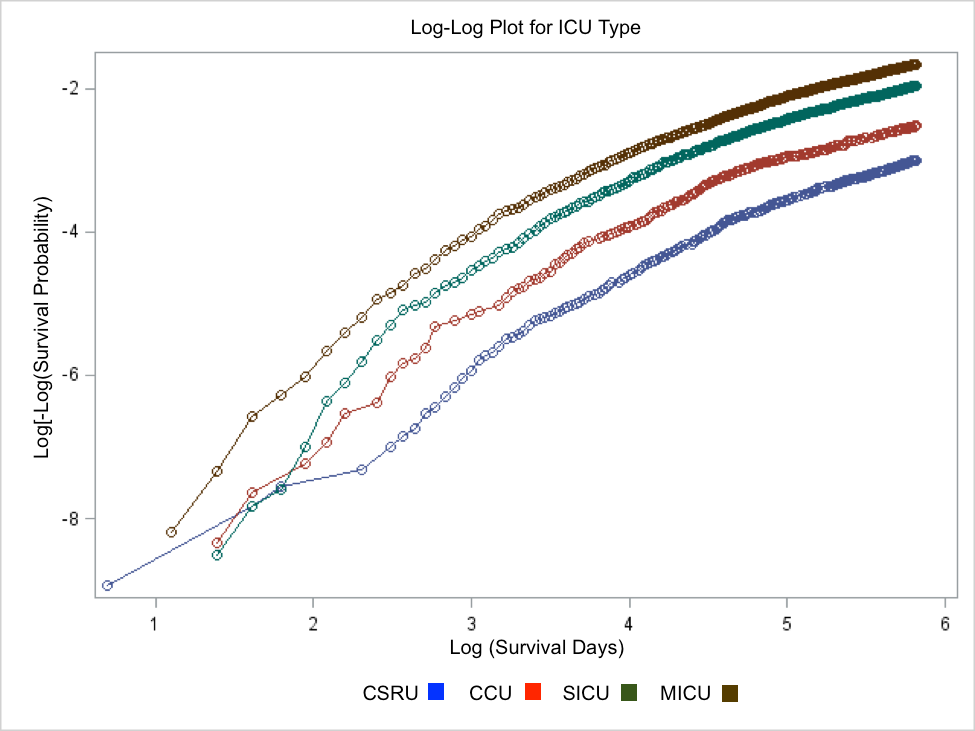

Supplement: S17 Fig — (PNG) [file pone.0197226.s019.png]

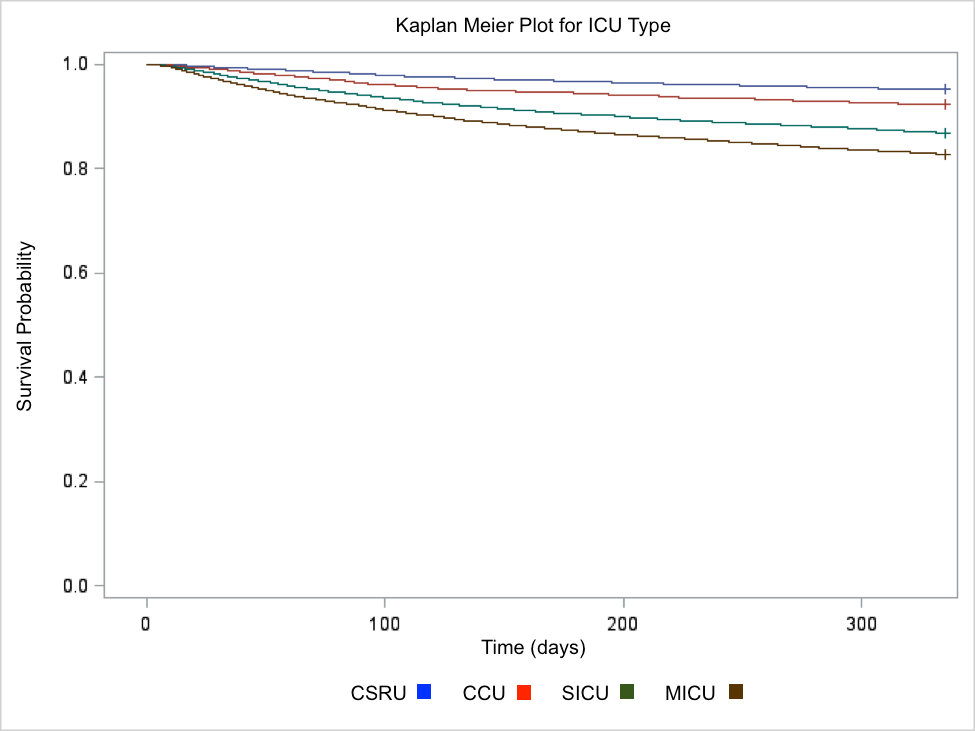

Supplement: S18 Fig — (PNG) [file pone.0197226.s020.png]

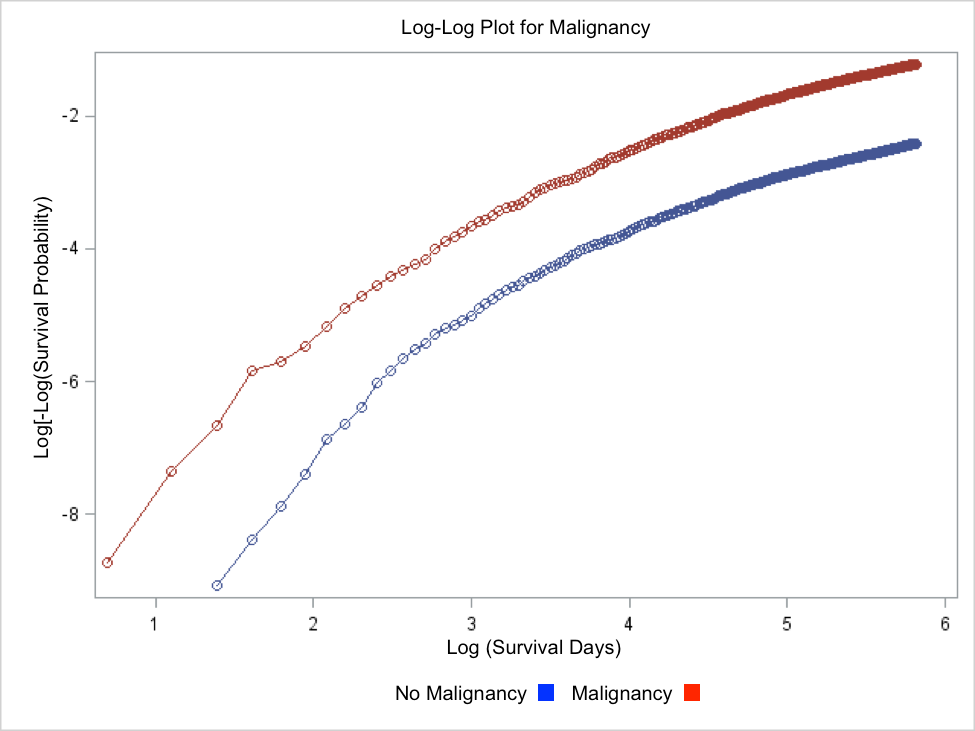

Supplement: S19 Fig — (PNG) [file pone.0197226.s021.png]

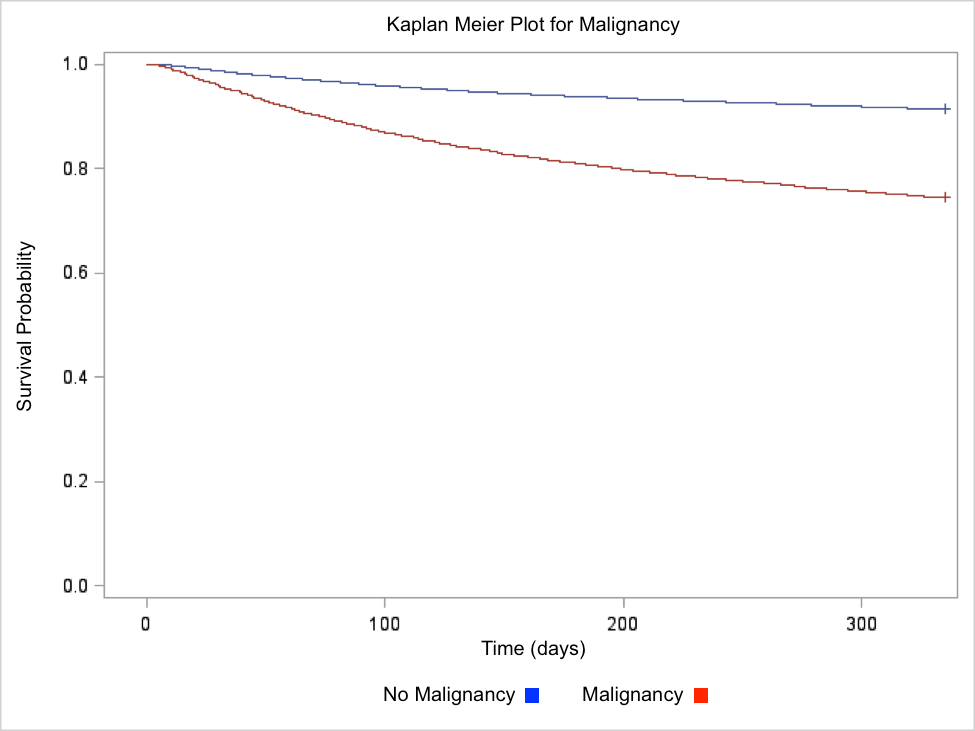

Supplement: S20 Fig — (PNG) [file pone.0197226.s022.png]

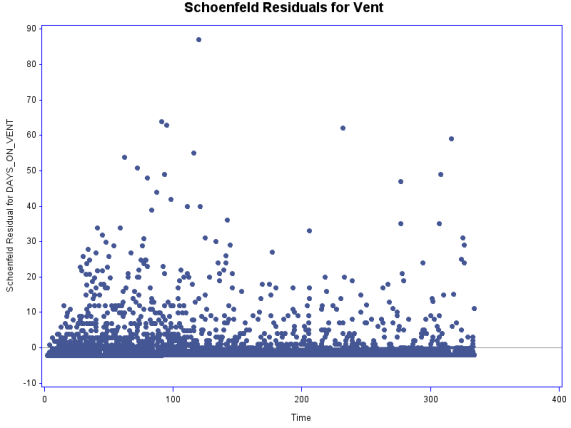

Supplement: S21 Fig — (PNG) [file pone.0197226.s023.png]

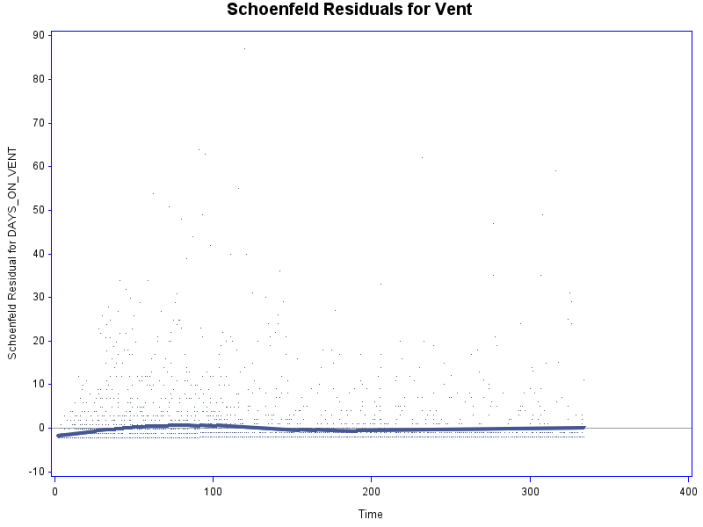

Supplement: S22 Fig — (PNG) [file pone.0197226.s024.png]

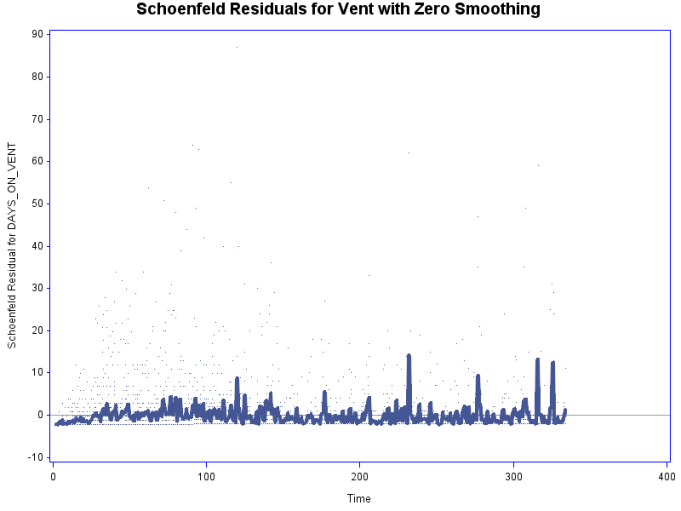

Supplement: S23 Fig — (PNG) [file pone.0197226.s025.png]
